# Circulating Endothelial Cells: A New Possible Marker of Endothelial Damage in Kawasaki Disease, Multisystem Inflammatory Syndrome in Children and Acute SARS-CoV-2 Infection

**DOI:** 10.3390/ijms231710106

**Published:** 2022-09-03

**Authors:** Marianna Fabi, Biljana Petrovic, Laura Andreozzi, Elena Corinaldesi, Emanuele Filice, Carlotta Biagi, Alessia Rizzello, Bianca Elisa Mattesini, Simone Bugani, Marcello Lanari

**Affiliations:** 1 Pediatric Emergency Unit, IRCCS Azienda Ospedaliero-Universitaria di Bologna, 40138 Bologna, Italy; 2 Department of Medical and Surgical Sciences (DIMEC), University of Bologna, 40138 Bologna, Italy; 3 Center for Applied Biomedical Research (CRBA), University of Bologna, 40138 Bologna, Italy; 4 Pediatric Unit, Ramazzini Hospital, 41012 Carpi, Italy; 5 Specialty School of Pediatrics, Alma Mater Studiorum, University of Bologna, 40138 Bologna, Italy; 6 Alma Mater Studiorum, University of Bologna, 40126 Bologna, Italy

**Keywords:** kawasaki disease, multisystem inflammatory syndrome in children, CellSearch, circulating endothelial cells, COVID-19, children, endothelial injury, coronary artery lesions

## Abstract

Background: Kawasaki Disease (KD) and Multisystem Inflammatory Syndrome in Children (MIS-C) are pediatric diseases characterized by systemic inflammation and vascular injury, potentially leading to coronary artery lesions (CALs). Data on vascular injury occurring during acute COVID-19 (AC19) in children are still lacking. The aim of our study was to investigate endothelial injury in KD-, MIS-C- and AC19-dosing circulating endothelial cells (CECs). Methods: We conducted a multicenter prospective study. CECs were enumerated by CellSearch technology through the immunomagnetic capture of CD146-positive cells from whole blood. Results: We enrolled 9 KD, 20 MIS-C and 10 AC19. During the acute stage, the AC19 and KD patients had higher CECs levels than the MIS-C patients. From the acute to subacute phase, a significant CEC increase was observed in the KD patients, while a mild decrease was detected in the MIS-C patients. Cellular clusters/syncytia were more common in the KD patients. No correlation between CECs and CALs were found in the MIS-C patients. The incidence of CALs in the KD group was too low to investigate this correlation. Conclusions: Our study suggests a possible role of CECs as biomarkers of systemic inflammation and endothelial dysfunction in KD and MIS-C and different mechanisms of vascular injury in these diseases. Further larger studies are needed.

## 1. Introduction

Kawasaki Disease (KD) is a pediatric acute self-limiting vasculitis affecting medium-sized vessels, with the possible involvement of coronary arteries (CA). The occurrence of coronary artery lesions (CALs) can heavily affect the outcome of this disease. In fact, in high-income countries, KD is the leading cause of pediatric-acquired heart disease [1]. KD should be suspected in children younger than five years old with persistent fever and distinctive clinical manifestations, including bilateral bulbar conjunctival injection, rash, cervical lymphadenopathy, extremity erythema and edema and oral mucosal changes in the acute phase [1]. A number of studies have previously shown a systemic inflammation characterized by increased inflammatory mediators that could lead to endothelial cell damage and dysfunction (ECD) after a still unknown environmental trigger [2,3]. Therefore, the optimal management requires an early diagnosis to vigorously reduce the inflammation in order to limit the ECD and potentially the development of CALs. In children with CALs, severe complications may occur such as ischemic heart disease and sudden death during either the acute or chronic phases of the disease [4]. In addition, since ECD is a recognized risk factor for atherosclerosis in adults, limiting ECD could modify the link between KD and atherosclerosis in later ages [5]. Treatment with intravenous immunoglobulin (IVIG) within the 10th day from fever onset significantly decreases the incidence of CALs [1] and adjunctive therapies could further improve the coronary outcome [6,7,8].

The COVID-19 pandemic has revealed a new pathological condition characterized by an abnormal inflammatory systemic response, the Multisystem Inflammatory Syndrome in Children (MIS-C), sharing clinical and laboratory features with KD and Kawasaki disease shock syndrome (KDSS). MIS-C usually develops 3–6 weeks following a SARS-CoV-2 infection with an incidence of 1 per approximately 3000 to 4000 infected children and adolescents [9,10,11]. Severe multisystem organ dysfunction, particularly cardiac injury, systemic shock and respiratory distress can affect the outcome of MIS-C, requiring Intensive Care Unit (ICU) admissions and massive support treatments. As KD, the prompt recognition of MIS-C is crucial in the attempt to halt the inflammation and thus the progression of organ damage, including the occurrence of CALs, myocardial systolic depression and hypotension or shock [12,13,14]. A cytokine storm triggered by a SARS-CoV-2 infection has been advocated as a potential basis of the disease leading to systemic vasculitis. Extremely high levels of matrix metalloproteinase 7 (MMP7), a protein involved in the degradation of endothelial junctions, potentially leading to vascular leak/edema and leukocyte migration into tissues, has been found in an adolescent girl with MIS-C [15]. Increased MMP7 has been previously also found in KD patients, too [15,16].

Acute COVID-19 (AC19) in adults is known to be associated with endothelial injury in different organs, such as the lungs, kidneys and small bowel [17,18]. The damage is probably mediated by the ACE-2-receptor, leading to cell swelling, the disruption of intercellular junctions and cell death [19,20]. Thrombotic complications have been reported in severe COVID-19 cases [21]. On the opposite, the acute infection is generally mild in children, including infants, with few recorded fatalities attributed to AC19 [22,23].

Therefore, these three diseases may share pathogenic mechanisms including systemic inflammation, vascular injury and endothelial dysfunction, leading to potentially life-threatening complications.

Since the structural and functional integrity of the endothelium is essential for the maintenance of vascular homeostasis, the detection of high levels of circulating endothelial cells (CECs) detached from blood vessels in the peripheral blood has been linked to endothelial derangement [24,25].

Adult healthy subjects carry low CECs counts, whereas high levels of CECs have been found in several adults’ conditions characterized by ECD, including infectious and cardiovascular diseases, inflammatory and connective tissue diseases, transplantation and cancer [26,27,28,29,30,31,32].

To date, there are very few studies investigating the role of CECs in children. In addition, the methods to study CECs are heterogeneous. Most of these studies focused on the close relationship between CECs and cardiovascular health in older children and adolescents with obesity, hypertension and other features of metabolic syndrome [33,34,35] where CECs have been correlated with adiposity and cardiometabolic risk factors, potentially reflecting an accelerated atherosclerosis. Moreover, CECs are higher in patients with sepsis and complicated sepsis than healthy controls [36], while the role of CECs is controversial in children with sarcomas [37,38].

Among the available methodologies for quantifying and isolating CECs, the CellSearch system represents the only procedure/methodology that guarantees a high level of standardization and specificity [32,39].

Our primary aim was to study CEC values as possible biomarkers of ECD in children diagnosed with KD, MIS-C and acute COVID-19.

The secondary aim was to investigate CECs as possible markers of disease severity in the same groups of patients, in order to identify children at higher risk early on, to modulate therapy and to prevent life-threatening complications.

## 2. Results

### 2.1. Patients

We enrolled a total of 39 patients, including 9 KD, 20 MIS-C and 10 AC19.

Demographic and clinical data are shown in Table 1.

During the acute phase, CALs were detected in only 1/9 (11.1%) of the KD patients and 8/20 (40%) of the MIS-C patients, half of which were aneurysms. CALs in the MIS-C patients showed a tendency to regress compared with the KD patients (see Table 2).

All the KD patients were treated with IVIG, while the MIS-C patients received steroids in addition to IVIG. Additionally, two KD patients, who were non-responders, received additional steroid therapy. Two KD patients and one MIS-C patient were administered Anakinra (an interleukin (IL)-1 antagonist) and one MIS-C patient was administered Infliximab (an anti-Tumor necrosis factor drug) when these patients did not respond to standard treatments. The AC19 patients did not receive any immunomodulatory/anti-inflammatory parenteral medication.

Laboratory values during the acute and subacute phases are shown in Table 3.

### 2.2. CECs Count at Acute and Subacute Stages

**Figure 1:**
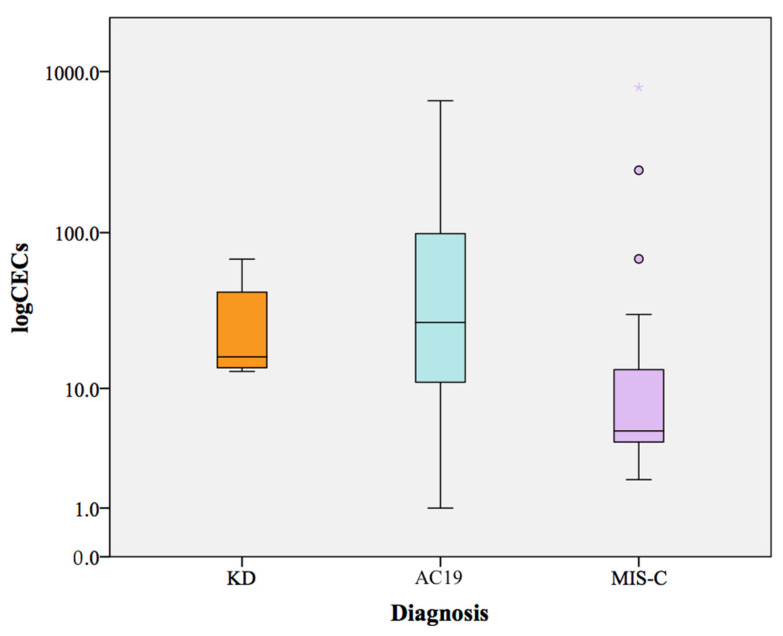
Boxplot of CEC levels in KD, AC19 and MIS-C patients during the acute stage of diseases. KD stands for Kawasaki Disease; MIS-C stands for Multisystem Inflammatory Syndrome in Children; AC19 stands for acute COVID-19.

The median CECs/mL count at acute stage in the KD patients was 16.3 (13.6–48.8 IQR) in comparison to 5 (4–15.5 IQR) and 27.1 (9.3–101.7 IQR) in the MIS-C and AC19 patients, respectively (Table 4, Figure 1). In three KD patients and one MIS-C patient, we were not able to collect CECs during the acute phase.

The AC19 patients showed a higher CEC count compared to the MIS-C and KD patients, without reaching a statistically significant difference because of high CEC count variability in the AC19 patients (ranging from 1 to 660 CECs/mL).

During the acute stage, the CEC median values were higher in the KD patients than in the MIS-C patients (*p* = 0.042). The median values of CECs in the MIS-C patients were within the normal range (1–14) reported in literature for healthy adult with the CellSearch system [31,40,41], whereas it was higher in the KD and AC19 patients.

**Figure 2:**
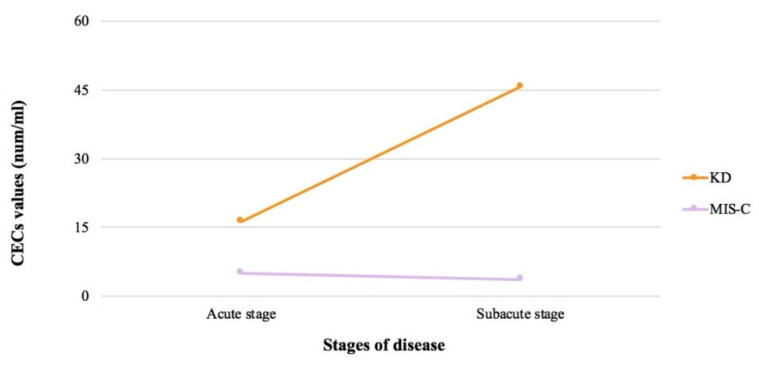
CECs trend from acute to subacute stage, in KD and MIS-C patients. KD stands for Kawasaki Disease; MIS-C stands for Multisystem Inflammatory Syndrome in Children; CECs stands for circulating endothelial cells.

During the subacute phase, a significant increase in the CEC values of the KD patients was observed, despite the difference between the two stages not being statistically significant (*p* = 0.11). On the contrary, a mild decrease in CECs was detected in the MIS-C patients from the acute to subacute stage. This different trend of the CEC values between the two stages of illnesses is shown in Figure 2. In the subacute phase, the CEC levels of KD were significantly higher than those of the MIS-C patients (*p* = 0.01). 

Age was inversely related to CEC values considering the whole sample of patients (*p* = 0.011). This finding was confirmed in the KD patients (*p* = 0.042) and a trend was found in the AC19 patients (*p* = 0.052).

We observed, in several samples, a CEC cell with a continuous cytoplasmic staining associated with two or more nuclei. This kind of cell aberration was previously described with the CellSearch procedure in acute myocardial infarction [32]. Since cell–cell boundaries are not clearly distinguishable in the fluorescence images, it is not possible to determine rigorously whether these are cellular clusters, or alternatively, multinuclear individual cells (syncytia).

However, these cellular clusters/syncytia were detected in 3/6 (50%) of the KD patients, 3/10 (30%) of the AC19 patients and 3/18 (16.7%) of the MIS-C patients during the acute stage. As expected, a positive correlation was found between CECs and the number of syncytia in the whole sample during the acute and subacute stage (*p* = 0.006 and *p* = 0.001, respectively) and in the KD + MIS-C cohort (*p* < 0.001).

### 2.3. CECs Count and Laboratory Values/Treatment

Considering the group KD+MIS-C, a significant positive correlation was found between CECs and fibrinogen in the acute stage (*p* = 0.032), steroid and heparin treatment (*p* = 0.003 and *p* = 0.007, respectively) and respiratory and cutaneous manifestations (*p* = 0.030 and 0.027, respectively). CECs were positively correlated with platelets [PLT] during the subacute stage (*p* = 0.018).

Considering the group MIS-C, a positive correlation between CECs and neutrophil percentage value [N%] (*p* = 0.043) and IL-10 (*p* = 0.014) was found.

No correlation was found between CECs values and IVIG responsiveness (*p* = 0.460).

### 2.4. CECs Count and Disease Severity

We did not find any correlation between CECs and the presence of CALs, including dilations and aneurysms, during acute and subacute stages, in the MIS-C patients. The incidence of coronary events in the KD group was too low to be investigated.

Cardiac non-coronary events were positively correlated with C-reactive protein [CRP] (*p* = 0.014), troponin (*p* = 0.023), brain natriuretic peptide [BNP] (*p* = 0.019) and N% (*p* = 0.04). Even though we could not find a direct correlation between CECs and cardiac events, the latter were positively related to N%, which was in turn correlated with CECs during the acute phase in MIS-C.

The persistence of CALs in the subacute phase was more likely to occur in IVIG non-responders (*p* = 0.012), in patients who needed biologics (*p* = 0.02) and who had a longer duration of fever (*p* = 0.014).

We did not find any correlation between CECs and clinical manifestations or a need for respiratory support in AC19, probably because the severity of AC19 was mild in all patients. In addition, the potential relation between CECs and respiratory or inotropic support was not explored in the KD patients because very few patients needed these additional therapies. Nonetheless, for the MIS-C patients, no significant correlation was found between CEC values and respiratory or inotropic support (*p* = 0.257 and *p* = 0.628, respectively).

## 3. Discussion

This is the first study measuring CECs in children with KD, MIS-C and acute COVID-19, and analyzing and comparing their trend over the duration of the diseases with the standardized procedure of the CellSearch system.

Our findings show a significant difference in CECs during the acute stage of the diseases; in particular, the MIS-C patients show the lower values. The difference becomes more significant during the subacute stage of the diseases, after the medical treatment, when a remarkable increase in CEC levels was observed only in the KD patients.

These findings may reveal a deep difference in the pathogenesis of KD and MIS-C, even though the possible vascular complications that may occur in the two diseases are similar (coronary artery dilations and aneurysms).

It is already known that necrotizing arteritis is the pathogenetic process responsible for the development of CALs during the acute stage of KD. The infiltration of CA by inflammatory cells leads to the disruption of collagen and elastin fibers and the loss of structural integrity, resulting in CA aneurysms and dilations [1]. In addition, ECD seems to persist years after the acute disease: Shah et al. found that CECs identified with CD146-immunomagnetic bead extraction were significantly higher in children with KD with and without CALs than healthy controls [42].

One of the steps of this process is the disruption of the endothelial layer, which may be responsible for the detachment of endothelial cells and subsequent high levels of CECs in peripheral blood, as found in our cohort [43]. This process is likely to explain the correlation found between CECs and CALs in KD by Nakatani et al., that we were not able to confirm probably due to the paucity of the CALs during the acute phase in our KD cohort [44].

Similarly, the low number of CECs found in the MIS-C patients during the acute stage may be an additional step toward a deeper understanding of the pathogenetic process involved in the development of CALs in these patients.

Although SARS-CoV-2 particles have been detected inside the endothelial cells and endothelial derangement has been found in children with MIS-C, the process responsible for CAL development may not include the loss of the integrity of the endothelial layer [13,45,46].

It has already been proved that in children diagnosed with juvenile idiopathic arthritis or non-KD febrile illnesses, CA dimensions may be larger than those in healthy afebrile subjects but smaller than the dimensions in KD patients [47]. Hence, a similar event may occur in MIS-C patients: CALs might be present due to high levels of circulating cytokines with subsequent endothelial cell dysfunction or edema leading to dilations of coronaries [46,48], rather than to a structural injury as it happens in KD.

This different pathogenesis of CALs might also explain the higher tendency of CALs to regress in the MIS-C patients than the KD patients, as already reported in previous studies and confirmed in ours [14].

The persistence of endothelial integrity in the MIS-C patients might contribute to explain the absence of an increase in the CECs levels in these patients during the acute stage, even in those with CALs.

The different trend of the CEC values between KD and MIS-C from the acute to subacute stage seems to support the hypothesis of different pathogenetic processes, highlighting the possible persistence of generalized long-term abnormalities of the systemic vascular structure and endothelial function even after the IVIG treatment in the KD patients. This trend was also in agreement with findings reported in a paper by Nakatani et al. [44], despite the CECs count being obtained with a different methodology.

It has already been proved that KD patients with either persisting or regressing CALs show an impaired long-term vascular function [49,50]. The persistence of high CEC levels during the subacute stage in patients without CALs, as documented in our cohort, may reveal a persistent endothelial dysfunction even in patients without coronary involvement in this stage.

On the other hand, the mild decrease in CEC levels in the MIS-C patients from the acute to subacute stage suggests a gradual resolution of the inflammatory process responsible for clinical symptoms and complications during the acute stage of the disease, with a complete healing of the vascular wall due to the absence of endothelial disruption. The treatment with steroids in addition to IVIG in these patients could accelerate the reduction in inflammation, with a rapid restoration of normal endothelial functions.

According to our findings, CEC levels may be used as an additional tool to distinguish KD from MIS-C at the onset of the disease, since these two conditions have a mostly clinical definition without pathognomonic features. Thus, they could also help to provide the proper treatment to patients and to define the time of the evaluation of possible complications.

The detection of clusters/syncytia in half of the KD patients, as opposed to the lower occurrence in the AC19 and MIS-C patients, may contribute to underlining the deep endothelial damage that distinguishes KD from AC19 and MIS-C. A syncytium is a multinucleate mass of cytoplasm resulting from the fusion of cells. Under normal conditions, fusion events are uncommon, but they increase in pathological conditions such as after tissue injury and during inflammation [51].

In addition, the detection of different levels of CECs with a persistent increase in KD, and MIS-C during the subacute stage, contributes to fill in some of the blanks on the influence of IVIG therapy on CECs [44]. Even though the KD and MIS-C patients both received IVIG infusion during the acute stage, the CEC levels were significantly different during the subacute stage, suggesting that the CEC number is not affected by IVIG therapy. On the other hand, further research is needed to clarify whether or not corticosteroid therapy affects CECs levels.

Moreover, a correlation with younger age and higher CEC levels was found in the whole sample and it was confirmed in the KD and AC19 cohorts. This finding could be related to the physiological deeper vascular remodeling potentially occurring in this age group.

In our cohort, CEC values in patients diagnosed with AC19 spanned a wide range of values, with high median levels. No correlation was found between CECs and clinical presentation in children diagnosed with AC19. CEC count and/or kinetics has never been investigated with the CellSearch system in adult COVID-19 patients. Studies performed with a validated flow cytometry procedure to enumerate CECs reported different, but not in contrast, results, depending on whether the absolute mature CECs count, viable/apoptotic mature CECs count or CEC progenitors, were evaluated. Mancuso et al. [52] found that the absolute count of mature CD146+ CECs was similar in healthy controls with respect to COVID-19 patients, but the viable/apoptotic CD146+ CEC ratio was significantly different. Nizzoli et al. reported that in 76.7% of COVID-19 patients, the mature CECs number was higher than a threshold of 30 CEC/mL; this percentage dropped to 16.7% in healthy controls [53].

There were some limitations in our study. First, the sample size was small, particularly for the KD cohort, due to the low incidence of KD in our country. In addition, in this particular group, the occurrence of coronary events was very low, so that it could partially explain the lack of correlation between CECs and CALs. On the contrary, a relatively high number of MIS-C patients were enrolled during a quite short time interval, due to the fact that our hospital is a tertiary referral center and due to the high incidence of COVID-19 cases during all the outbreaks in our region.

Second, CellSearch cannot distinguish CECs and endothelial progenitor cells (EPCs), which are thought to originate from the bone marrow and to circulate through the bloodstream in order to reach the sites of endothelial injury and repair the damage [54]. Additionally, consensus on the definition of EPCs has not been achieved yet and the phenotypic differentiation of EPC and CEC is still lacking [24,54].

Third, a bias in the trend of CECs through the subacute phases may have occurred, due to the more aggressive immunomodulatory therapy received by the MIS-C patients compared to the KD patients, which could more effectively limit inflammation and subsequent endothelial damage.

## 4. Materials and Methods

### 4.1. Patients

We conducted a multicenter prospective study including all children diagnosed with KD and MIS-C between October 2020 and June 2021 in 2 Pediatric Units (IRCCS-St. Orsola University Hospital and Ramazzini Hospital) in Emilia-Romagna (Italy) and all children diagnosed with AC19 admitted at the IRCCS-St. Orsola Hospital Pediatric Emergency Department (PED) in the same time interval. The study was conducted according to the guidelines of the Declaration of Helsinki and was approved by the local Ethics Committee (Comitato Etico Area Vasta Emilia Centro—AVEC, Bologna, Italy; protocol codes: No. 95/2021/Sper/AOUBo; No. EM44-2021_340/2017/O/Oss/AOUBo). Written informed consent was obtained from the parents.

All KD diagnoses and treatments were made in accordance with the 2017 American Heart Association (AHA) Guidelines [1].

MIS-C diagnoses were made according to WHO criteria, including clinical, laboratory and microbiological features, in patients with evidence of a SARS-CoV-2 infection or who were a likely contact with confirmed cases [55].

For both KD and MIS-C, the onset of illness was defined as the first day of fever. The time interval between the disease onset and the 10th day of fever was defined as the “acute phase”, while the one between the 11th and the 20th day after the fever onset was defined as the “subacute phase”.

Considering the responsiveness to IVIG treatment, patients were divided into responders and non-responders. IVIG non-responsiveness was defined as the persistence or recrudescence of fever at least 36 h and less than 7 days after completion of the first IVIG infusion. Late treatment was defined for KD when IVIG was given after the 10th day of fever.

Echocardiography was performed in all children diagnosed with KD and MIS-C at each participating center at diagnosis and between the 11th and 20th day after diagnosis. CALs were classified as ectasia and aneurysms according to their z-score criteria, as recommended by AHA KD guidelines [1]. The evolution of CALs from the acute to subacute stage was recorded as a binary variable, including 2 possibilities: persistence or regression.

AC19 was diagnosed in patients that tested positive for SARS-CoV-2 RT-PCR in nasopharyngeal swab samples and that reported symptoms compatible with COVID-19. The onset of illness was defined as the day when the first symptom or sign occurred.

A database was prospectively created and subsequently retrospectively reviewed. It included demographic features and clinical manifestations (respiratory symptoms, conjunctival hyperemia, extremity changes, skin rash, oral changes, cervical lymphadenopathy, abdominal involvement, systemic hypotension/shock), date of diagnosis, length of hospital stay, time and response to treatment (IVIG responders, IVIG non-responders, late treated) and laboratory values (white blood cells [WBC]; N%, lymphocyte [L%] and eosinophil [E%] percentage values; neutrophils–lymphocytes ratio [NLR]; red blood cells [RBC]; hemoglobin [Hb]; PLT; CRP; serum albumin; alanine aminotransferase [ALT]; sodium [Na], albumin, troponin, BNP; IL-10) recorded within the 10th day of illness. Laboratory data of the KD and MIS-C patients were also collected during the subacute phase.

Abdominal involvement was defined as the presence of vomit and/or diarrhea and/or abdominal pain.

Severe disease was defined as development of CALs or the occurrence of cardiac non-coronary events or the requirement of respiratory support/inotropic therapy.

### 4.2. CellSearch System

CEC counts were performed by means of the Circulating Endothelial Cell Kit in combination with the CellSearch system (Menarini Silicon Biosystems, Castel Maggiore, Bologna, Italy), which allows one to standardize the whole procedure of the cellular selection, monoclonal antibodies labelling, analysis and enumeration of CECs as described previously [39].

Briefly, a blood sample of 4 mL is mixed with a ferrofluid-based capture reagent and immunofluorescent reagents. The ferrofluid reagent consists of nanoparticles with a magnetic core surrounded by a polymeric layer coated with antibodies targeting the CD146 antigen to capture CECs. After immunomagnetic capture and enrichment, fluorescent reagents, which include anti-CD105-PE, anti-CD45-APC and DAPI, are added. To be scored as CECs, a CD146+ cell must have a nucleus (DAPI), express CD105, have the morphology of an intact cell and be negative for CD45. Therefore, a CEC is defined as a CD146+/CD105+/DAPI+/CD45- cell. The results are expressed as the number of CECs per milliliter of peripheral blood. Occasionally, we observed a continuous CD105 cytoplasmic staining associated with two or more nuclei. The CECs were enumerated during the acute (before standard treatment) and subacute stages in children with KD and MIS-C.

### 4.3. Statistical Analysis

The normality of the continuous variables was assessed by the Kolmogorov–Smirnov normality test. The continuous variables are presented as the mean ± standard deviation (SD) or median and interquartile range (IQR), as appropriate. The continuous normally distributed variables were compared using the *t* test or ANOVA; non-parametric data were compared using the Mann–Whitney U test or Kruskal–Wallis test. For the categorical variables, the percentage of patients in each category was calculated and compared with chi-square or Fisher’s exact test, when appropriate. The matched-pairs Wilcoxon signed rank test and Friedman test were used to test statistical significance for the within-subject analysis. The level of statistical significance was set at *p* < 0.05. The analysis for this study was performed with SPSS Statistics software (version 25; SPSS Inc., Chicago, IL, USA).

## 5. Conclusions

In conclusion, our findings suggest a possible role of CECs as biomarkers of systemic acute inflammation and endothelial dysfunction in KD and MIS-C patients. These findings contribute to the differential diagnosis of KD and MIS-C and suggest a different etiopathogenesis of these diseases.

Additionally, CECs could be considered as a potential new tool for the identification of different physiopathological mechanisms of endothelial injury, thus guiding the therapeutic management of these pediatric systemic inflammatory conditions. In addition, CECs could represent a marker to monitor endothelial status also after the acute stage. Further larger studies are needed to confirm our findings.

## Figures and Tables

**Table 1:** Demographic and clinical data of the three groups of patients: KD, MIS-C and AC19.

		KD (n = 9)	MIS-C (n = 20)	AC19 (n = 10)	*p*
Ethnicity, n (%)	Caucasian	8 (88.9%)	18 (90%)	8 (80%)	*n.s.*
	Asian	1 (11.1%)	0 (0%)	2 (20%)	*n.s.*
	Black	0 (0%)	2 (10%)	0 (0%)	*n.s.*
Sex, n (%)	Male	3 (33.3%)	11 (55%)	5 (50%)	*n.s.*
	Female	6 (66.7%)	9 (45%)	5 (50%)	*n.s.*
Age in months, median (IQR)		22 (7.4–29) *	95 (69.8–115.6) *	26.5 (10.5–157.8)	***<0.001***
Respiratory symptoms, n (%)		1 (11.1%)	6 (30%)	5 (50%)	*n.s.*
Conjunctivitis, n (%)		9 (100%) *†	12 (60%) *‡	1 (10%) †‡	***<0.001***
Extremity changes, n (%)		5 (55.6%)	6 (30%)	1 (10%)	*n.s.*
Rash, n (%)		9 (100%) *†	11 (55%) *‡	1 (10%) †‡	***<0.001***
Oral changes, n (%)		6 (66.7%)	12 (60%)	3 (30%)	*n.s.*
Cervical lymphadenopathy, n (%)		7 (77.8%) *†	3 (15%) *	2 (20%) †	***0.002***
Abdominal involvement, n (%)		6 (66.7%)	17 (85.0%) ‡	2 (20%) ‡	***0.002***
Hypotension, n (%)		0 *	11 (55%) *‡	0 *‡	***0.001***
Total days of fever, median (IQR)		9.0 (5.5–13.0)	6.0 (4.0–9.0)	-	*n.s.*
Length of stay, median (IQR)		8.0 (6.5–15.0)	10.0 (8.0–13.0)	-	*n.s.*
Day of standard treatment, median (IQR)		8.5 (5.3–9.0) *	5.0 (4.0–6.0) *	-	***0.006***
Non-responders, n (%)		3 (33.3%)	5 (25%)	-	*n.s.*
Inotropic therapy, n (%)		1 (11.1%) *	11 (55%) *	-	***0.017***
Respiratory support, n (%)		0	5 (25%)	0	*n.s.*

KD stands for Kawasaki Disease; MIS-C stands for Multisystem Inflammatory Syndrome in Children; AC19 stands for acute COVID-19; * stands for statistically significant difference between KD and MIS-C; † stands for statistically significant difference between KD and AC19; ‡ stands for statistically significant difference between MIS-C and AC19. n.s. stands for not significant.

**Table 2:** Coronary involvement in KD and MIS-C groups during acute and subacute phases.

	KD (n = 9)	MIS-C (n = 20)	*p*
**Acute phase**			
CALs, n (%)	1 (11.1%)	8 (40%)	*n.s.*
*Aneurysms, n (%)*	*1 (100%)*	*4 (50%)*	*n.s.*
Non-coronary cardiac events, n (%)	2 (22.2%)	15 (75%)	***0.030***
**Subacute phase**			
CALs, n (%)	2 (22.2%)	1 (5%)	*n.s.*
*Aneurysms, n (%)*	*2 (100%)*	*1 (100%)*	*n.s.*
Non-coronary cardiac events, n (%)	0 (0%)	3 (15%)	*n.s.*

KD stands for Kawasaki Disease; MIS-C stands for Multisystem Inflammatory Syndrome in Children; CALs stands for coronary artery lesions; n.s. stands for not significant.

**Table 3:** Laboratory values during the acute and subacute phases of children diagnosed with KD, MIS-C and AC19.

**Acute Phase**				
	**KD (n = 9)**	**MIS-C (n = 20)**	**AC19 (n = 10)**	***p***
Hb g/dl, median (IQR)	11.8 (10.8–12.0)	10.8 (10.0–12.0)	12.3(11.2–12.5)	*n.s.*
RBC ×10^12^/L, median (IQR)	4.2 (4.0–4.8)	4.0 (3.7–4.5)	4.5 (3.9–4.8)	*n.s.*
PLT ×10^9^/L, median (IQR)	338 (325–397) *	151 (124–263) *	312 (244–334)	***0.003***
WBC ×10^9^/L, median (IQR)	14.4 (11.3–16.7) †	9.0 (6.3–13.1)	8.1 (5.5–11.3) †	***0.026***
N%, median (IQR)	71.4 (68.9–79.6)	78.2 (73.7–86.5) ‡	44.1 (24.5–59.2) ‡	***<0.001***
L%, median (IQR)	18.9 (11.5- 22.9)	13.1 (9.6–20.5) ‡	41.1 (30.1–63.8) ‡	***<0.001***
NLR, median (IQR)	4.1 (3.1–7.0)	6.0 (3.6–8.9) ‡	1.1 (0.4–2.3) ‡	***<0.001***
E%, median (IQR)	0.7 (0.4–2.6)	0.2 (0.1–1.6)	2.1 (0.6–2.5)	*n.s.*
CRP mg/dL, median (IQR)	10.7 (5.1–17.1)	17.6 (11.6–22.0) ‡	0.5 (0.1–3.7) ‡	***<0.001***
PCT, ng/mL, median (IQR)	0.8 (0.3–3.5) *	13.9 (3.3–39.5) *	0.8 (0.8–0.8)	***0.047***
Albumin g/dL, median (IQR)	3.4 (3.0–3.8) *	3.2 (3.0–3.8) *‡	4.6 (4.1–4.8) ‡	***0.002***
Na mmol/L, median (IQR)	135 (133–138)	134 (131–136) ‡	138 (136–143) ‡	***0.005***
ALT IU/L, median (IQR)	23 (14–177)	27 (18–85)	16 (14–29)	*n.s.*
BNP pg/mL, median (IQR)	139 (91–202)	450 (57–1090)	-	*n.s.*
IL-10 pg/mL, median (IQR)	5.5 (2.3–9.0)	8.0 (2.8–34.5)	-	*n.s.*
**Subacute stage**				
	**KD (n = 9)**	**MIS-C (n = 20)**		***p***
Hb g/dL, median (IQR)	10.7 (9.5–12.2)	11.1 (10.1–11.8)		*n.s.*
RBC ×10^12^/L, median (IQR)	3.9 (3.6–4.5)	4.1 (3.6–4.3)		*n.s.*
PLT ×10^9^/L, median (IQR)	695 (459–823)	391 (268–571)		***0.018***
WBC ×10^9^/L, median (IQR)	13.3 (9.6–15.5)	12.7 (9.7–16.0)		*n.s.*
N%, median (IQR)	46.0 (28.4–55.6)	71.5 (53.3–76.3)		***0.012***
L%, median (IQR)	46.3 (32.6–60.5)	24.4 (19.1–35.9)		***0.001***
NLR, median (IQR)	1.0 (0.5–1.9)	2.9 (1.5–4.1)		***0.004***
E%, median (IQR)	1.9 (0.7–5.5)	0.2 (0.1–0.9)		***0.021***
CRP mg/dl, median (IQR)	1.0 (0.5–6.9)	1.4 (0.8–1.9)		*n.s.*
PCT, ng/mL, median (IQR)	4.1 (0.2–4.1)	0.8 (0.3–3.8)		*n.s.*
Albumin g/dL, median (IQR)	3.6 (3.2–3.8)	3.7 (3.3–4.2)		*n.s.*
Na mmol/L, median (IQR)	138 (137–141)	139 (136–140)		*n.s.*
ALT IU/L, median (IQR)	25 (16–44)	37 (22–60)		*n.s.*
BNP pg/mL, median (IQR)	24 (14–39)	81 (38–140)		***0.042***

KD stands for Kawasaki Disease; MIS-C stands for Multisystem Inflammatory Syndrome in Children; AC19 stands for acute COVID-19; * stands for statistically significant difference between KD and MIS-C; † stands for statistically significant difference between KD and AC19; ‡ stands for statistically significant difference between MIS-C and AC19; Hb stands for hemoglobin; RBC stands for red blood cells; PLT stands for platelets; WBC stands for white blood cells; NLR stands for neutrophils–lymphocytes ratio; N% stands for neutrophil percentage values; L% stands for lymphocyte percentage values; E% stands for eosinophil percentage values; CRP stands for C-reactive protein; Na stands for sodium; ALT stands for alanine aminotransferase; BNP stands for brain natriuretic peptide; IL stands for interleukin; n.s. stands for not significant.

**Table 4:** CEC values during acute and subacute stages, in the three groups of patients.

	KD (n = 9)	MIS-C (n = 20)	AC19 (n = 10)	*p*
**Acute Phase**				
CECs num/mL, median (IQR)	16.3 (13.6–48.8) *	5 (4–15.5) *	27.1 (9.3–101.7)	***0.042***
CECs > nv, n (%)	6/6 (100%) *	5/19 (26.3%) *‡	7/10 (70%) ‡	***0.003***
Syncytia, n (%)	3/6 (50%)	3/19 (15.8%)	3/10 (30%)	*n.s.*
**Subacute phase**				
CECs num/mL, median (IQR)	45.8 (18.5–131.0) *	3.6 (1.8–21.6) *	-	***0.01***
CECs > nv, n (%)	7/9 (77.8%) *	6/18 (30%) *	-	***0.046***
Syncytia, n (%)	4/9 (44.4%)	3/18 (16.7%)	-	*n.s.*

KD stands for Kawasaki Disease; MIS-C stands for Multisystem Inflammatory Syndrome in Children; AC19 stands for acute COVID-19; CECs stands for circulating endothelial cells; * stands for statistically significant difference between KD and MIS-C; ‡ stands for statistically significant difference between MIS-C and AC19; nv stands for normal values; n.s. stands for not significant.

## Data Availability

The data presented in this study are available upon request from the corresponding author.
